# Factors associated with hygiene practices among primary school children in southern Ethiopia

**DOI:** 10.3389/fpubh.2024.1402455

**Published:** 2024-11-01

**Authors:** Eyasu Bamlaku Golla, Dawit Denano Leta, Alegntaw Abate, Habtamu Geremew, Samuel Abdisa Kuse

**Affiliations:** ^1^College of Health Science, Oda Bultum University, Chiro, Ethiopia; ^2^School of Public Health, College of Health Science and Medicine, Wolaita Sodo University, Soddo, Ethiopia; ^3^Department of Medical Laboratory, College of Health Science, Oda Bultum University, Chiro, Ethiopia

**Keywords:** primary school, hygiene practices, associated factors, water, sanitation, and hygiene (WASH), Ethiopia

## Abstract

**Background:**

Eight years into the Sustainable Development Goal period, Ethiopia is not on track to achieve good hygiene practices among school children. Ensuring good hygiene practices among primary school children to prevent the spread of communicable diseases remains a challenge in most primary schools in Ethiopia. Therefore, the aim of this study was to identify factors associated with hygiene practices among primary school children in southern Ethiopia.

**Methods:**

A school-based cross-sectional study was conducted from June 3 to July 28, 2022, in five primary schools. The simple random sampling technique was used to select the school. Subsequently, 640 students were selected from the proportionally allocated sample size. Pretested semi-structured interviewer-administered questionnaires and observational checklists were utilized to collect data. The data was then entered into EpiData version 4.6 and analyzed using SPSS version 25. Variables with a *p* ≤ 0.25 at bivariate analysis were used to develop a multivariable logistic model to identify factors associated with hygiene practices. *P* < 0.05 with a 95% confidence interval was considered statistically significant.

**Results:**

The magnitude of overall good hygiene practices was 29.2% (95% CI: 25.81–32.59). Urban residence (AOR = 3.4, 95% CI 2.1–5.55), knowledge of handwashing (AOR = 4.5, 95% CI 2.8–7.36), being a member of a hygiene and sanitation club (AOR = 3.7, 95% CI 2.4–6.86), and experience of visiting a model school (AOR = 3.1, 95% CI 2.1–5.55) were found to be significantly associated with hygiene practices.

**Conclusion:**

The overall level of good hygiene practices in Kedida district was low. Therefore, it is essential to enhance health education on handwashing, establish sanitation and hygiene clubs, and conduct visits to district model elementary schools.

## Introduction

Hygiene describes a range of activities performed to maintain health and prevent the spread of diseases. It includes key hygiene practices such as hand washing, latrine utilization, and safe water-handling practices (1). Good hygiene practice is very important in schools to avoid infectious diseases because the occurrence and severity of hygiene-related outbreaks amongst primary school children are greatly enhanced by school children's hygiene practice behavior (1, 2). If feces are disposed of and hands are washed with soap at critical times, the spread of disease can be reduced by 30% and 40%, respectively (2, 3). As a result of this, the importance of promoting hygiene practices has been endorsed in many international policies, yet many school children still have poor hygiene practices (3).

The burden of hygiene-related impacts remains a significant public health concern globally. Despite investments into hygiene practices yielding numerous achievements in developed nations, ~2.6 billion people have poor hygiene practices, with the majority residing in developing nations. The most vulnerable subgroup of the population is rural primary school children (4). According to a World Health Organization report in 2016, only 30.3% of primary school children worldwide were observed to be using proper hygiene practices, while the rest were reported to have improper hygiene practices. Due to unsafe water, sanitation, and hygiene (WASH) practices, nearly 700 school children die from diarrheal diseases every day. The highest burden of this issue is observed in developing countries, indicating that school children in these nations are bearing the consequences of poor hygiene practices (5, 6).

In Africa, the level of hygiene practices varies across different settings. Good hygiene practices were observed among 69% of school children in South Africa and 58% in Eswatini. Conversely, hygiene practices were reported to be very poor in countries such as Madagascar, Mozambique, Namibia, and Ethiopia. Open defecation was also noted as a common practice in the rural areas of these nations (6–9).

In sub-Saharan Africa, governments and other stakeholders, such as the WHO and the United Nations Children's Fund (UNICEF), have made several efforts to improve hand hygiene in schools (7). But a rapid growth in urbanization combined with a lack of essential infrastructure and hygiene facility improvement have limited the capacity of most African nations to provide adequate hygiene practice services to school children, which leads to inadequate school hygiene practices (6, 10, 11).

The Sustainable Development Goals (SDGs) set an ambitious vision to achieve universal access to “safely managed” water and sanitation (including hygiene), with a focus on helping every child gain access to clean drinking water and sanitation and hygiene facilities including in schools and health centers and in humanitarian situations when children are most vulnerable. But, 8 years into the SDG period, Ethiopia is not on track to achieve school hygiene. Achieving good hygiene practices among school children in Ethiopia requires a fourfold increase in current rates of progress on good hygiene practices (5, 12, 13).

In Ethiopia, reports have noted that the problem of hygiene practices is significantly high, contributing to 60% of communicable diseases and many child deaths each year. This issue significantly undermines children's ability to learn and impairs physical development. Additionally, due to this burden, many schoolchildren are absent from school. Although the health sector primarily focuses on modeling schools in health, achieving performance in Ethiopia is challenging. Therefore, further research is needed to identify factors associated with students' hygiene practices.

Factors significantly associated with hygiene among school children vary considerably among schools in Ethiopia (14–21). However, the aforementioned studies on primary schools were based on interviewer-administered questionnaires and did not involve the authors observing hygiene facilities such as the availability of water, soap, and toilets. The lack of these details hinders the application of relevant improvements. Furthermore, in contrast to the present study, previous studies were conducted on a very small number of schools, mostly in urban areas, indicating that local data is crucial for developing appropriate interventions, especially for both urban and rural primary school children. The district health office's annual report showed that intestinal parasites, diarrhea, and respiratory tract infections were among the top diseases affecting children under five. The present study also addresses an information gap regarding hygiene practices among urban and rural primary schools in Kedida district, southern Ethiopia. Therefore, the aim of this study was to identify the determinants of hygiene practices among primary school children in southern Ethiopia.

## Methods and materials

### Study setting

The study was conducted in Kedida district, one of the districts in southern Ethiopia, located 271 km away from Addis Ababa, the capital city of Ethiopia. According to the 2007 Census conducted by the CSA, the district has a total population of 89,391 (44,589 men and 44,802 women). The district has 27 primary schools, with six schools teaching grades 5 and 6, three schools teaching grades 5, 6, and 7, and the remaining 16 primary schools teaching grades 5 to 8. In the academic year 2021/22, a total of 6,448 primary school children (2,842 females and 3,606 males) were enrolled in all primary schools, with 2,352 males and 2,390 females in the second cycle of primary school. The district Health Office's 2020 annual report indicated that latrine coverage, latrine utilization, and drinking water coverage were 55%, 61%, and 45%, respectively (22).

### Study design and period

A school-based cross-sectional study was conducted from June 3 to July 28, 2022.

### Population

All children in the district's primary schools were the source population and the study population was all children who attended primary school in five randomly selected schools.

### Inclusion and exclusion criteria

All grade 5–8 students from the academic year 2021/2022 in five randomly selected primary schools in the district were included in the study, while students who were absent during data collection were excluded from the study.

### Sample size determination

The sample size was estimated using a single population proportion formula, namely (Zα/2)2 ^*^ p(1-p)/d^2^, assuming a 95% confidence interval, 80% test power, a design effect of two, a 10% non-response rate, and a proportion of good hygiene practice among primary school children at 30.4% (P1 = 30.4%) based on previous studies (15). Initially, 715 individual students were recruited. However, since the population was less than ten thousand (10,000), a correction formula was applied, resulting in a revised sample size of 640. The sample size was calculated with the following assumption (refer to Table 1) and then 640 was taken as our final sample size.

**Table 1:** Sample size determination for associated factors with hygiene practice, 2022.

**Associated factors**	**95% CI**	**Power (*B)***	**% proportion**	**AOR**	**Sample size**	**final n**	**Reference**
Knowledge of water handling	95%	80%	33.4	2.99	138	151.8	(12)
Knowledge of hand washing	95%	80%	40.8	2.28	233	256.3	(10)
Mainly uses the latrine	95%	80%	28.9	2.03	330	363	(10)
Ever having visited a model school	95%	80%	34.8	2.44	201	221.1	(13)

### Sampling technique

**Figure 1:**
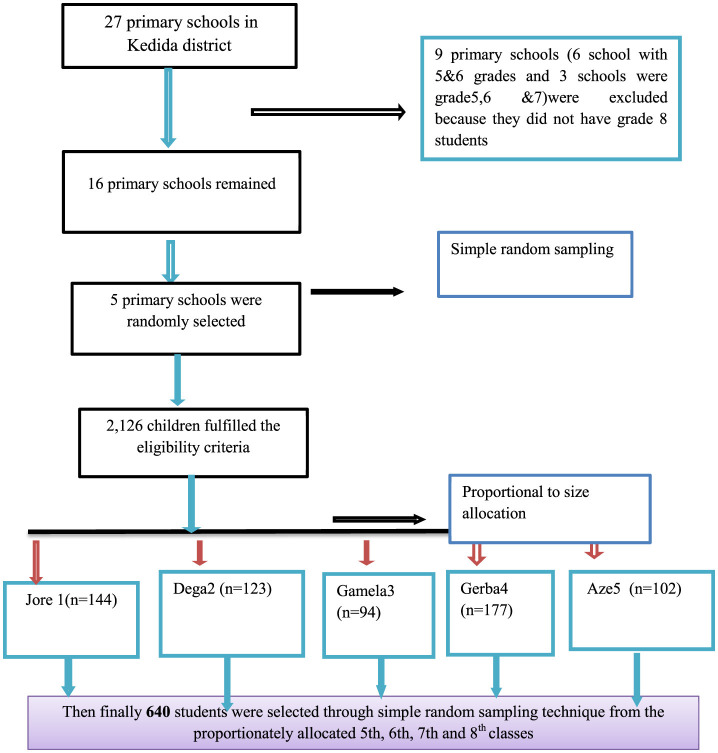
Sampling presentation for the assessment of hygiene practices and associated factors among primary school children in Kedida district, south Ethiopia, 2022 (*n* = 640).

A multi-stage probability sampling procedure was used to select participating students. In the district, there were 27 primary schools, of which 16 schools taught students in grades 5–8 in 2022/2023. From these 16 primary schools, five were selected using a simple random sampling technique by listing all schools as a sampling frame. The sample size was then allocated proportionally to the size of each selected school. Subsequently, 640 students were selected through simple random sampling from the proportionately allocated classes (Figure 1).

### Operational definitions

#### Hygiene practice

“Hygiene refers to conditions and practices that help to maintain health and prevent the spread of diseases” (4). According to this study, hygiene practice was assessed using 16 hygiene practice indicator questions, which were adapted from the WHO's three key hygiene practice-indicators and from similar studies conducted in Ethiopia (3, 15). From these, four items involved water handling practice-indicators, four items involved latrine utilization practice-indicators, and eight items involved hand-washing practice-indicators.

Students who scored ≥65 out of sixteen questions and ≥10 overall on practice indicator items were categorized as having good hygiene practice and those who failed to score at least 65% were categorized as having poor hygiene practice (13, 15, 16).

#### Knowledge about hygiene practice

A child is classified as having good knowledge on water handling if he/she answered “yes” to at least three of five questions, good knowledge on latrine utilization if he/she answered “yes” to at least two of four questions, and good knowledge on hand washing if students knew at least three out of the five critical times of hand washing practice and answered “yes” to four of six other question items (15, 16).

#### Good water-handling practice

Students who “always” clean and cover drinking water containers and do not touch drinking water with dirty hands were classified as having good water-handling practice (15).

#### Latrine utilization

For this item, it was necessary for there to be functional latrines with no observable feces in the compound and at least two signs of latrine use from observation (fresh feces in the pit, a visible footpath to the latrine, wet slab, a smelly latrine, and visibly used anal-cleansing material) (9, 15).

#### Data collection procedures

Data were collected through a semi-structured questionnaire and observational checklists which were developed after reviewing relevant literature (1, 15–21). The questionnaire and observational checklist were first prepared in English and then translated into the local language, Kembahatissa, before being translated back to English for consistency. Four health extension workers were recruited as data collectors and two teachers were involved as the data collection facilitator and supervisor, respectively, in the school setting. Data collectors and supervisors were trained for two days about the data collection process.

A pretest from 5% of the total sample size was conducted in nearby district primary schools that had the same socio-demographic characteristics as those of the study schools. Based on the pretest, questions were revised and edited and the necessary corrections were made accordingly. Latrine utilization was verified through observation of at least two signs of latrine use. Each interviewee was given a unique identification code, which is used during data entry. Completeness was checked every day.

### Data processing and analysis

Data was entered into EpiData version 4.6 statistical software and then exported to SPSS version 25.0 for analysis. Once exported to SPSS, simple frequencies, distribution, percentage, mean, and standard deviation were computed to describe the data. Binary logistic regression analysis was then performed to identify factors associated with hygiene practices through crude and adjusted odds ratios. Initially, bivariable logistic regressions were conducted to assess the crude association of each independent variable and to select candidate variables for multivariable analysis. Variables with a *p* ≤ 0.25 from the bivariable analysis were chosen as candidate variables for multivariable logistic regression. Subsequently, a multivariable logistic regression (adjusted odds ratio) was conducted to identify independent predictors of hygiene practices. The presence of multicollinearity among independent variables was assessed using the variance inflation factor (VIF), which indicated no multicollinearity (1 <VIF <2). Model fitness was evaluated using the Hosmer–Lemeshow goodness-of-fit test, showing a good fit (*P* = 0.0724, i.e., > 0.05). Adjusted odds ratios were calculated with a 95% confidence interval and variables with a *p*-value <0.05 were considered statistically significant factors. Finally, the study findings were presented through text, tables, and graphs accordingly.

### Ethical consideration

Before actual activities, ethical clearance was obtained from Wolaita Sodo University College of Medicine and Health sciences ethical review committee on Jun 26, 2022 (*Ref. no: SGS/124/22*). A support letter was written from (23) Kedida district education office to all selected primary schools on Jun 30, 2022 (*Ref. no:* ቀ/ት/*212/1/9*). Informed assent was obtained from parents/guardians (on behalf of school children) after fully explaining the purpose of the study. Written informed consent from parents/guardians was collected 2 days before data collection via their children. Administrative permissions were taken from school directors. The information was kept confidential and anonymous. The study had no risk and/or direct benefit to study subjects. The right of the respondents to withdraw from the interview or not to participate was respected.

## Results

### Sociodemographic characteristics of the respondents

From a total of 640 respondents, 634 were involved in the study, resulting in a total response rate of 99.06%. Additionally, approximately 328 (51.7%) of the participants were male, and 306 (48.3%) were rural dwellers. Similarly, the majority of the respondents (408, 64.4%) were from grades 5–6, while the lowest number of participants were from grades 7–8, totaling 225 (35.6%). The median age of the respondents was 16 years (Table 2).

**Table 2:** Sociodemographic characteristics of respondents in Kedida district, South Ethiopia, 2022 (*n* = 634).

**Variables**	**Categories**	**Frequency**	**Percentage**
Age	10–14	315	49.7
	15–19	319	50.3
Sex	Male	328	51.7
	Female	306	48.3
Mother's educational status	Unable to read and write	224	35.3
	Primary	171	27
	Secondary	146	23
	College and above	93	14
Residence	Rural	340	53.6
	Urban	294	46.4
Father's educational status	Unable to read and write	232	36.6
	Primary	215	33.9
	Secondary	110	17.4
	College and above	77	12.1
Family size	2–4	271	42.7
	5–10	243	33.3
	Above 10	120	18.9
Grade of the student	5–6	408	64.4
	7–8	225	35.6
Parent's occupation (head)	Farmer	224	35.3
	Merchant	220	34.7
	Government employee	114	18
	Other	76	12

### Observation findings

Observation was conducted in five selected primary schools. All of them have separate latrines for female and male students, as well as for staff members. The availability of latrines in each school was three (60%) in coverage, but upon observing utilization, only two (40%) were properly utilized. In terms of water availability, four schools (80%) have different types of water sources. However, when it comes to water handling and utilization, only one (20%) school properly utilizes their water supply.

Most of the observed students were not used to using water for anal cleansing in their schools. None of the schools had soap available to wash hands in the school washing basin. The classrooms were not cleaned, and there was no planned sanitation campaign for the schools.

### Magnitude of hygiene practice among school children

**Figure 2:**
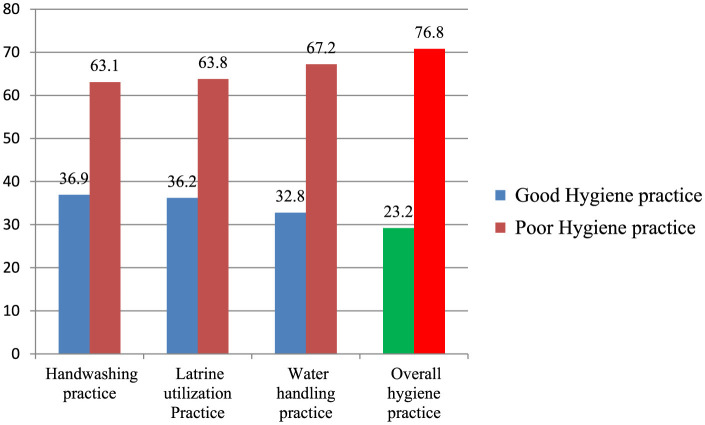
Hygiene practices among primary school children in Kedida district, South Ethiopia, 2022. Red: Poor practice. Blue: Good practice. Green: Good hygiene practice. Bright red: Poor hygiene practice.

The overall magnitude of good hygiene practice in the study area was 185(29.2%: 95% CI: 25.81–32.59) (Figure 2).

### Factor associated with hygiene practice

After applying multivariable logistic regression analysis on the final model, being an urban dweller (AOR = 3.4 95% CI 2.1–5.55), having knowledge of hand washing (AOR = 4.5 95%CI 2.8–7.36), being a member of a hygiene and sanitation club (AOR = 3.7 95%CI 2.4–6.86), and experience of visiting a model school (AOR = 3.1 95%CI 2.1-5.55) were found to be significantly associated with good hygiene practice with *p* < 0.05 (Table 3).

**Table 3:** Binary logistic regression analysis of factors associated with hygiene practice among primary school children in Kedida district, 2022 (*n* = 634).

**Variables**	**Categories**	**Hygiene practice**		**COR (95 % CI)**	**AOR (95 % CI)**
		**Good**	**Poor**		
Sex	Female	101 (30.4%)	231 (69.6%)	1.14 (0.8–1.6)	
	Male	84 (27.8%)	218 (76.2%)	1	
Age	15–19	97 (31%)	221 (69%)	1.1 (0.82–1.63)	
	10–14	88 (28%)	228 (72%)	1	
Grade	2.7–8	96 (54.2%)	75 (45.8%)	3.4 (3.8–7.8)^*^	1.4 (0.96–2.9)
	1.5–6	89 (19%)	374 (81%)	1	1
Residence	1. Urban	123 (44.7%)	152 (55.3%)	3.9 (2.7–5.6)^*^	3.4 (2.1–5.55)^*^
	2. Rural	62 (17.3%)	297 (82.7%)	1	1
Family size	1.2–4	120 (39.3%)	185 (61.7%)	0.357 (0.27– 0.47)	
	2.5–10	55 (26.7%)	151 (74.3%)	1	
	3. Above 10	10 (8.1%)	113 (91.9%)		
Occupation of parents	1. Farmer	52 (17.4%)	247 (82.6%)	1.537 (1.29–1.82)	
	2. Merchants	37 (19.3%)	155 (80.7%)		
	3. Govt employees	77 (58.1%)	3 (3.8%)	1	
	4. Others	9 (17.3%)	43 (82.7%)		
Knowledge of hand washing practice	1. Good	140 (53%)	124 (47%)	8 (5.5–12)	4.5 (2.8–7.4)^*^
	2. Poor	45 (12%)	325 (88%)	1	1
Knowledge of water handling	1. Good	120 (56.6%)	92 (43.4%)	7 (5.0–10.5)^*^	1.2 (0.88–4.3)
	2. Poor	65 (15.4%)	357 (84.6%)	1	1
Knowledge of latrine utilization	1. Good	91 (51%) 94	87 (49%) 362	2.0 (2.8–5.9)^*^	1.8 (0.98–3.8)
	2. Poor	(20.6%)	(79.4%)	1	1
Participating in Hygiene and sanitation club	1. Yes	85 (31.3%)	143 (62.7%)	4.8 (3.1–6.6)^*^	3.7 (2.4–5.9)^*^
	2. No	100 (24.7%)	306 (75.3%)	1	1
Visiting model school	1. Yes	83 (39.3%)	128 (60.7%)	3.3 (2.2–4.7)^*^	3.1 (1.9–4.4)^*^
	2. No	102 (24.2%)	321 (75.8%)	1	1
School latrine	1. Yes	102 (43.8%)	168 (56.2%)	2.0 (1.45–2.908)^*^	1.7 (1.01–2.8)
	2. No	83 (22.8%)	281 (77.2%)	1	1
Source of information about hygiene	1. School club	293 (67.1%)	87 (33.95%)	4.96 (2.505–8.371)	
	2. Hex worker	153 (61.7%)	94 (39.3%)		
	3. Radio/TV	1 (33.3%)	2 (67.3%)		
	4. Others	2 (40%)	3 (60%)	1	

^*^p <0.05 (significantly associated variables, adjusted).

## Discussion

This study was conducted to identify the magnitude of overall hygiene practices and to determine the factors associated with them among primary school children. The study found that the prevalence of good hygiene practices was 29.2% (95% CI: 25.81–32.59). This result aligns with studies conducted in Merako district (30.4%) (15), Wolaita zone, Ethiopia (28.1%) (18), and Sebata town, Ethiopia (32.8%) (21).

Our result was higher than those in studies conducted in Arbaminch town, Ethiopia, which was 22.3% (14), and Sudan (22.85%) (24). However, the finding was lower than studies conducted in Mereb-Lake District, Ethiopia (61.7%) (19), Harar, Ethiopia (37%) (20), Bihar, India (44.9%) (25), China (42.05%) (26), and Colombia (36.6%) (27). The possible reasons for this variation in proportion might be the results of sociodemographic, economic, and behavioral differences; increased access to handwashing facilities both in schools and homes; and facility provisions and promotional activities regarding the effectiveness of handwashing due to the COVID-19 pandemic in the above studies. Another explanation for this might be due to a lack of knowledge on hygiene practices in the study area (41.25%).

Good water handling practices among primary school children in Kedida District were found to be 32.8%, which is significantly lower than results in a similar study conducted in Mereb-Leke District, which achieved 83% (19). This disparity could be attributed to inadequate sanitation facilities, children touching drinking water with dirty hands (53%), and the presence of drug users (54.9%).

Good latrine utilization practice in our study was found to be 40%. This is much lower than the rates of 91% in Mehal Meda town in Amhara (27), 67% in Loma District, Ethiopia (9), and 56% at the federal level in Ethiopia (28). The lower percentage in our study could be attributed to variations in study time and area, inadequate hygiene facilities, the presence of unimproved latrines, and drug users. Good handwashing practice was also found to be 37%, which is lower compared to rates of 73.8% in Yirgalem (29), 58.9% in Mereb-Leke District (19), and 76% in Angola (30). This difference may be due to variations in the study area, inadequate hygiene facilities, lack of water and soap/ash, and the fact that some children wash their hands without soap/ash at critical times (17.2%).

The findings of this study indicate that 43%, 42.3%, and 43.8% of children had sufficient knowledge of handwashing, latrine utilization, and water handling practices. This percentage is lower than the findings of studies conducted in Yirgalem (60%) (29) and Mereb-Leke District (65%) (19). This discrepancy can be attributed to inadequate access to hygiene facilities and geographical variations. The study also shows that students' knowledge increased, leading to a relatively high proportion of hygiene practices among those with adequate knowledge.

Results from the logistic regression analysis show that the odds of having good knowledge of hand washing was 4.5 times higher than having poor knowledge of hand washing, the odds of having good knowledge of latrine utilization were 2.3 times higher than having poor knowledge of latrine utilization, and the odds of having good knowledge of water handling was 2.6 times higher than having poor knowledge of water handling. The finding that knowledge was associated with hygiene practice was supported by studies conducted in Mereb-lake Ethiopia (19), Angola (30), and Bangladesh (3). Moreover, that a lack of hygiene facilities may lead to lower hand washing practice, latrine utilization practice, water handling practice, and overall hygiene practice was also supported in other studies (26–28).

In this study, there were different sociodemographic factors affecting the overall hygiene practices of school children. Among those, significant predictors of hygiene practices of primary school children included being an urban dweller (AOR = 3.4, 95% CI 2.1–5.55) and being in a higher grade level (AOR = 4.0, 95% CI 2.4–6.86), which were positively associated with hygiene practices. Younger learners were less likely to show significant differences in their views and participation in hygiene and sanitation. This is because higher-grade learners tend to be more knowledgeable and cautious about hygiene and sanitation issues. Additionally, another study also indicates that students in higher grades were more aware than those in lower grades, as knowledge tends to increase as children progress from lower to higher grades (18, 20, 31–34).

According to this study, residence is one of the sociodemographic factors that is associated with overall hygiene practice. Hygiene practice was different in rural and urban settings. Urban dwellers practice hygiene better than rural residents. This could be due to the fast delivery of information to urban students, high levels of acceptance of national initiatives like Handwashing Days by urban primary school children, and further intervention by health workers and school sanitation. This may increase the frequency and compliance of students to hygiene practices in urban areas compared to rural areas. The results of this study showed that the odds of having good hygiene practices by students in urban areas were 3.4 times higher than in students living in rural areas (AOR=2.57, 95% CI 1.269–5.208). This finding was consistent with studies conducted in Ethiopia, Ghana, and India (21, 35–37).

### Limitation of the study

A cross-sectional study might not be strong enough to determine a direct cause-and-effect relationship. We were not able to control confounders, such as a household's hygiene facilities and wealth index, which might have contributed to hygiene practice. This study did not include observations of students practicing hygiene activities in their home and it also did not include their parents' opinion about their children's hygiene practice in the home. Behavioral theories outline structural and psychological processes that can control human behavior and might be essential for changing behavior. But we did not address these behavioral theories within this article.

### Conclusion and recommendations

According to the water and sanitation target of the World Health Organization (WHO), the overall level of good hygiene practices in Kedida district was low. This study found that knowledge of handwashing, being a member of a hygiene and sanitation club, and visiting model schools were significantly associated with hygiene practices. Therefore, the Kedida district education office, health office, school WASH program, and various NGOs should focus on and collaborate with schools to enhance the knowledge of school children on proper handwashing. This can be achieved by establishing and actively engaging in hygiene clubs, and teachers should be encouraged to visit model schools. Health extension workers and teachers should raise awareness about students' hygiene practices by providing effective health promotion and disease prevention education. We further recommend addressing the most important facilitators and barriers toward adequate practice through behavioral theories and models.

## Data Availability

The raw data supporting the conclusions of this article will be made available by the authors, without undue reservation.
